# A Case Report on Metformin-Associated Lactic Acidosis

**DOI:** 10.7759/cureus.9533

**Published:** 2020-08-03

**Authors:** Harpreet K Rai, Pranavkumar Patel, Kalpana Reddy

**Affiliations:** 1 Internal Medicine, Northwell Health Long Island Jewish Forest Hills Hospital, Forest Hills, USA; 2 Endocrinology, Northwell Health Long Island Jewish Forest Hills Hospital, Forest Hills, USA

**Keywords:** metformin, lactic acidosis, hemodialysis, acidosis, diabetes, severe acidosis, acidemia

## Abstract

Metformin is the first-line therapy for patients with type 2 diabetes, and its most common adverse effects are gastrointestinal. Lactic acidosis associated with metformin use is rare. Here, we report the case of a 77-year-old man with a medical history of diabetes (treated with metformin), hypertension, chronic alcohol abuse, and prostate and bladder cancer, who presented with abdominal pain, nausea, vomiting, and diarrhea for five days. He was admitted with severe metabolic acidosis due to metformin toxicity (metformin-associated lactic acidosis) with metformin level 23 mcg/mL (therapeutic range approximately 1-2 mcg/mL) in the setting of acute kidney failure due to acute pancreatitis and sepsis secondary to aspiration pneumonia. He was intubated, required pressor support, and received daily hemodialysis. Despite aggressive management, his hospital course became complicated with acute respiratory distress syndrome, myocardial infarction, acute hepatic failure, and ischemic and metabolic encephalopathy. In the end, the family decided to withdraw care and the patient was terminally extubated.

## Introduction

Metformin is the principal biguanide, which became available for clinical use in the 1950s and was introduced into the United States in 1995 [1]. Metformin is mainly absorbed in the upper small intestine, circulates unbound in the plasma, and is eliminated unchanged by the kidneys. The therapeutic range of metformin levels in the plasma is <2 mcg/mL [2]. Metformin plasma levels >5 mcg/mL are generally found when metformin is implicated as the cause of lactic acidosis [3]. Such high levels of metformin in the plasma can result from an acute overdose, reduced clearance of metformin due to poor kidney function, reduced lactate clearance in impaired hepatic metabolism, and/or increased production of lactate seen in conditions such as sepsis or anoxia.

Based on the plasma metformin levels and presence of any underlying condition that can cause lactic acidosis, lactic acidosis in metformin therapy can be categorized as follows: metformin-induced lactic acidosis (MILA), metformin-associated lactic acidosis (MALA), and metformin-unrelated lactic acidosis (MULA) [4]. MILA includes the cases where there is absence of any known condition that can lead to lactic acidosis and there is high plasma levels of metformin (e.g., in acute metformin overdose). MALA occurs when patients currently treated with metformin develop an acute life-threatening illness (e.g., septic shock or cardiogenic shock). In MALA, metformin amplifies the lactic acidosis, but it is not the sole cause of the illness. MULA occurs when lactic acidosis is present, but metformin is an innocent bystander and its levels are low.

MALA is extremely rare, with an estimated incidence of 0.03 to 0.06 per 1,000 patient-years based on spontaneous case reports [5]. Most cases occurred in patients with predisposing conditions or intercurrent precipitating events that predispose to lactic acidosis [5].

We present the case of a 77-year-old man with a medical history of diabetes treated with metformin and admitted with severe MALA in the setting of acute kidney failure due to acute pancreatitis and sepsis due to aspiration pneumonia.

## Case presentation

A 77-year-old man with a medical history significant for type 2 diabetes, hypertension, chronic alcohol abuse, and prostate and bladder cancer presented to the hospital with abdominal pain, nausea, vomiting, and diarrhea lasting for five days. His home medications included metformin 1,000 mg twice a day, sitagliptin 100 mg once a day, glipizide 10 mg once a day, lisinopril 2.5 mg once a day, and metoprolol succinate 50 mg once a day. His last primary care physician follow-up was nine months prior to presentation, and at that time, his baseline creatinine was 1.2 mg/dL (normal range: 0.50-1.30 mg/dL). On arrival to the emergency department, he was afebrile with a blood pressure of 180/78 mmHg, heart rate 94 beats per minute, respiratory rate 20 breaths per minute, and 100% saturation on room air. The patient was in moderate distress due to abdominal pain. His physical examination was significant for diffuse abdominal tenderness. Laboratory findings were remarkable with a white blood cell count of 11.13 K/µL (normal range: 3.80-1.50 K/µL), lipase of 1,597 U/L (normal range: 73-393 U/L), sodium level of 131 mmol/L (normal range: 135-145 mmol/L), potassium of 5.4 mmol/L (normal range: 3.5-5.3 mmol/L), and bicarbonate of 15 mmol/L (normal range: 22-31 mmol/L). His anion gap was 18 mmol/L (normal range: 5-17 mmol/L), blood urea nitrogen was 55 mg/dL (normal range: 7-18 mg/dL), creatinine was 7.39 mg/dL, and estimated glomerular filtration rate (eGFR) was 6 mL/min/1.73 m^2^. His albumin level was 3.7 g/dL (normal range: 3.5-5.0 g/dL), lactate was 9.7 mmol/L (normal range: 0.7-2.0 mmol/L), and his blood glucose level was 23 mg/dL. A CT scan of his abdomen showed no acute abdominal pathology but was significant for bilateral lower lung opacities. Blood and urine cultures were sent for evaluation. He was admitted with alcohol-induced acute pancreatitis, sepsis due to aspiration pneumonia, and acute kidney failure. Metformin toxicity was a concern in the setting of acute kidney failure secondary to gastrointestinal losses due to acute pancreatitis. His metformin levels were tested. He received aggressive intravenous (IV) fluid resuscitation and was started on piperacillin-tazobactam.

Within four hours of admission, the patient developed acute respiratory distress. A chest X-ray showed severe bilateral infiltrates concerning pulmonary edema and pneumonia. The patient was started on noninvasive ventilation and was transferred to the intensive care unit for close monitoring. His respiratory status continued to worsen, requiring endotracheal intubation and pressor support for a distributive shock from sepsis and sedative medication. His repeat laboratory evaluation results were significant for lactate of 13.1 mmol/L, bicarbonate of 8 mmol/L, anion gap of 26 mmol/L, and arterial pH of 6.94 (normal range: 7.35-7.45). The patient was in severe metabolic acidosis due to metformin toxicity (i.e., MALA) in the setting of acute kidney failure due to acute pancreatitis and sepsis. The patient was started on bicarbonate drip and had emergent hemodialysis. Despite mechanical ventilation and hemodialysis, his respiratory status continued to worsen, requiring high FiO_2_ and positive end-expiratory pressure. Acute respiratory distress syndrome was a concern, so he was paralyzed with cisatracurium. The patient had a repeat dialysis session on day 2, to prevent refractory lactic acidosis and for fluid overload. After the second session of dialysis, his lactate levels improved to 3.8 mmol/L, creatinine decreased to 3.49 mg/dL, anion gap decreased to 16 mmol/L, arterial pH improved to 7.2, and bicarbonate improved to 18 mmol/L.

Over the next few days, his hospital course was complicated with supraventricular tachycardia, non-ST elevation myocardial infarction, and acute liver failure. He continued to receive daily dialysis. His blood pressure and oxygen requirement started to improve. The sedation was tapered off on day 9, but he did not have any mental status. A CT scan of the head showed preserved white gray matter differentiation in the cortex and was negative for any acute pathology. An electroencephalogram showed generalized slowing related to ischemic and metabolic encephalopathy. Given the inability to wean the patient from the ventilator due to poor mental status and dependence on hemodialysis, his family decided to withdraw care. The palliative team was involved; the patient was terminally extubated and transferred to inpatient hospice. The patient died on day 14 of hospitalization. Later, the metformin levels on admission were revealed as 23 mcg/mL (the therapeutic concentration is 1-2 mcg/mL).

## Discussion

Previously metformin use was contraindicated at serum creatinine levels of 1.4 mg/dL or higher in women and 1.5 mg/dL or higher in men. However, in a recent update, the US Food and Drug Administration (FDA) target eGFR as a better estimate of renal function of the patient [6]. FDA concluded that metformin is safe in those with an estimated eGFR greater than 45 mL/min/1.73 m^2^ and is contraindicated in those with eGFR less than 30 mL/min/1.73 m^2^. The FDA recommends not initiating metformin for patients with eGFR between 30 and 45 mL/min/1.73 m^2^, but it also recommends annual monitoring of eGFR in all patients taking metformin. In patients at increased risk for the development of renal impairment such as the elderly, renal function should be assessed more frequently. In patients taking metformin whose eGFR later falls below 45 mL/min/1.73 m^2^, assess the benefits and risks of continuing treatment. 

MALA is more likely to occur in patients who acutely develop renal impairment from dehydration, vomiting, or diarrhea, especially in elderly subjects who have a reduced glomerular filtration rate [5]. According to some estimates, approximately 25% of patients taking metformin have one or more contraindications [7]. 

Lactic acidosis resulting from metformin toxicity should be suspected in any patient meeting all of the following criteria: a history of metformin administration; a markedly elevated lactate level (>15 mmol/L) with a large anion gap (>20 mmol/L); severe acidemia (pH 7.1); a very low serum bicarbonate level (<10 mmol/L); and a history of renal insufficiency (glomerular filtration rate <45 mL/min or serum creatinine level >2.0 mg/dL) [8].

Our patient had a serum creatinine of 1.2 mg/dL (which corresponds to an approximate eGFR of 60 mL/min/1.73 m^2^ or chronic kidney disease stage 2), nine months prior to his presentation to the hospital. He developed acute kidney failure due to gastrointestinal losses associated with acute pancreatitis and sepsis. Lactic acidosis was amplified due to metformin use; therefore, he presented critically ill. There is no antidote for metformin. Severe metabolic acidosis may be treated with hemodialysis irrespective of the underlying causes. Also, metformin level testing is not available in all the hospitals and obtaining metformin levels is often a lengthy process. Clinicians should be mindful of the possibility of MALA in patients presenting with lactic acidosis due to other causes like sepsis. Metformin is a commonly used drug and the incidence of MALA may still be underdiagnosed. 

The mainstay of treatment for MALA is supportive care. Mechanical ventilation may not always be necessary in all cases of MALA. There is no right choice for IV fluids. Normal saline is an acidotic fluid, and lactated ringer is not indicated as these patients are not able to metabolize lactate. The use of bicarb drip is controversial in metformin toxicity [9]. Also, raising the pH with bicarbonate may stimulate glycolysis and, thereby, increase lactate generation. Hemodialysis is indicated for profound acidosis with pH < 7.1, highly elevated lactate (>15-20 mmol/L), and failure to improve despite standard supportive measures. Comorbid conditions such as shock and liver failure may lower the threshold for dialysis. Hemodialysis will correct metformin-induced acid-base disturbance and slightly increase the clearance of metformin. Hemodialysis should be performed using a bicarbonate buffer, as the benefits of hemodialysis mainly lie in correcting acidosis rather than removing metformin. Hemodialysis may be discontinued when the lactate concentration is <3 mmol/L and the pH is >7.35 [10]. 

Our patient received aggressive treatment with mechanical ventilation, pressor support, and daily hemodialysis; yet he died. MALA is associated with high mortality. In a case series of 49 metformin-treated patients with lactic acidosis, mortality was 45% [11].

## Conclusions

We present a relatively rare but significant complication of the commonly used drug metformin-MALA. Physicians should use caution when using metformin to treat older patients with mild renal insufficiency. Such patients are at high risk of developing MALA due to acute illnesses such as gastroenteritis, sepsis, and acute heart failure. Kidney function should be closely monitored in these patients. It is important to educate the patients on the risk of MALA, and patients should be advised to stop taking the medication and seek urgent medical care in case of any acute illness that may increase the risk of MALA. Clinicians should be mindful of the possibility of MALA in patients presenting with severe lactic acidosis due to other causes such as sepsis. As there is no antidote for metformin, and treatment of severe metabolic acidosis mainly relies on hemodialysis, the incidence of MALA may still be underdiagnosed.
